# High Diversity and Prevalence of Potentially Pathogenic Free-Living Amoebae in Water Sources from Castilla y León, Spain

**DOI:** 10.3390/pathogens14070637

**Published:** 2025-06-25

**Authors:** Patricia Pérez-Pérez, Iván Rodríguez-Escolar, José E. Piñero, Rodrigo Morchón, Jacob Lorenzo-Morales

**Affiliations:** 1 Instituto Universitario de Enfermedades Tropicales y Salud Pública de Canarias (IUETSPC), Universidad de La Laguna (ULL), 38206 San Cristóbal de La Laguna, Spain; pperezpe@ull.edu.es (P.P.-P.); jpinero@ull.edu.es (J.E.P.); 2 Departamento de Obstetricia y Ginecología, Pediatría, Medicina Preventiva y Salud Pública, Toxicología, Medicina Legal y Forense y Parasitología, Universidad de La Laguna, 38200 San Cristóbal de La Laguna, Spain; 3 Zoonotic Infections and One Health GIR, Laboratory of Parasitology, Faculty of Pharmacy, University of Salamanca, 37007 Salamanca, Spain; ivanrodriguez@usal.es (I.R.-E.); rmorgar@usal.es (R.M.); 4 Biomedical Research Institute of Salamanca (IBSAL), University of Salamanca, 37007 Salamanca, Spain; 5 Centre for Environmental Studies and Rural Dynamization (CEADIR), University of Salamanca, 37007 Salamanca, Spain; 6 Centro de Investigación Biomédica en Red de Enfermedades Infecciosas (CIBERINFEC), Instituto de Salud Carlos III, 28029 Madrid, Spain

**Keywords:** Castilla y León, Spain, water, *Acanthamoeba* spp., *Vermamoeba vermiformis*, monitoring

## Abstract

Free-living amoebae (FLA) such as *Acanthamoeba* spp., *Balamuthia mandrillaris*, *Naegleria fowleri*, *Sappinia pedata*, *Vermamoeba vermiformis* and *Vahlkampfia* spp. are causal agents of deadly and/or disabling infections in humans. Despite recent data showing an increase in infection cases worldwide, studies on the prevalence of these emerging pathogens in water sources are scarce. Moreover, climate change is believed to facilitate the expansion and persistence of these environmental pathogens, further emphasizing the need for comprehensive surveillance. Therefore, the current study investigates the variety and abundance of free-living amoebae in different water sources in the autonomous community of Castilla y León, Spain, during different seasons of the year. *Vermamoeba vermiformis* was the most prevalent species and was detected in rivers, swamps, irrigation waters, swimming pools and recreational fountains. Moreover, genera such as *Acanthamoeba* and *Naegleria* and *Vahlkampfia* were also identified. This study highlights the diversity of FLA in the region and their relationship with local water characteristics. Given that certain FLA species are opportunistic pathogens, these results emphasize the necessity of monitoring this area and water sources.

## 1. Introduction

Free-living amoebae (FLA) mostly exist in the environment such as water, soil, or dust without the requirement for a host [1,2]. Under favorable conditions, FLA adopt the form of trophozoites or the vegetative stage, in charge of feeding and multiplication. When external conditions are adverse, FLA turn to the cyst form or resistant stage, surrounding themselves with a highly robust wall to protect them against environmental hazards [3].

Among the many FLA genera found in nature, infections in humans and other animals are caused by *Acanthamoeba* spp., *Naegleria fowleri*, *Sappinia pedata*, *Balamuthia mandrillaris*, *Vermamoeba vermiformis*, and *Vahlkampfia* spp. [1,4,5]. Many of these amoebae cause brain infections, e.g., *Acanthamoeba* species, *B. mandrillaris*, and *S. pedata*, produce granulomatous amoebic encephalitis (GAE), whereas *N. fowleri* produces primary amoebic meningoencephalitis (PAM) [1,6,7]. However, *Acanthamoeba* spp., *V. vermiformis* and *Vahlkampfia* spp. are also causative agents of amoebic keratitis [8]. A recent study found that *V. vermiformis* is the cause of a painful ulcer next to the eye and has been found to be both an etiological agent and a pathogen reservoir [9]. Additionally, *B. mandrillaris* and *Acanthamoeba* species cause skin disorders in immunocompromised individuals [1].

FLA are increasingly considered as environmental vehicles of other microorganisms. In addition, it is important to mention that FLA primarily feed on other microorganisms, especially bacteria, through phagocytosis. In fact, several bacteria species have acquired resistance mechanisms to the FLA digestive enzymes (amoeba-resistant bacteria, ARB), using FLA as vehicles. Moreover, the cyst stage can facilitate the intracellular survival of bacteria, avoiding common water disinfection systems or agents, but they are not effective against FLA cysts [10,11]. As a result, FLA contribute to the recycling of nutrients in freshwater settings, which makes them important to the ecology of many ecosystems [12].

In Spain, research on FLA has grown due to their impact on public health. However, studies have been conducted mainly in the Canary Islands and have contributed significantly to the detection of these parasites in the environment [13,14,15,16]. On the other hand, researchers from the University of Zaragoza analyzed FLA in water and sludge from five wastewater treatment plants (WWTP) in the Ebro River basin [17] and other authors studied the presence of FLA during a year in water from four drinking water treatment plants (DWTP), seven wastewater treatment plants (WWTP) and six locations of influence (LI) on four river basins from Spain [18]. In general, these studies are essential to assess public health risks, especially in contexts of direct human exposure, to monitor environmental impacts and to design prevention and control measures. Additionally, there is no specific regulation in Spain on FLA in water, unlike other countries such as Mexico, Australia and France, which have limited the number of *N. fowleri* cells (100 cells/L), indicating a gap in the environmental control of these pathogens [19,20].

Therefore, due to the lack of knowledge about the environmental prevalence of FLA in Spain, particularly in a Continental Mediterranean climate, the aim of the present work was to increase the epidemiological knowledge of FLA through one year of sampling in different water sources located in the autonomous community of Castilla y León.

## 2. Materials and Methods

### 2.1. Location and Sampling

**Figure 1:**
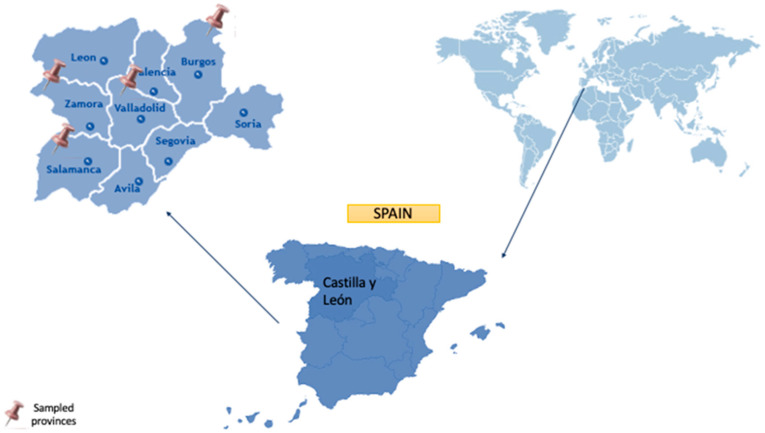
Geographical localization of the autonomous community of Castilla y León.

The sampling area was in the autonomous community of Castilla y León, covering the north-central part of the Iberian Peninsula in an elevated plateau (~800 m a.s.l.), surrounded by three mountain systems in the north, south and east. Castilla y León presents an area of 94,224 km^2^, and it is the largest region in Spain and one of the largest in Europe. The autonomous community is divided into 9 provinces: León, Zamora, Salamanca, Valladolid, Palencia, Burgos, Soria, Segovia, and Ávila (Figure 1). According to Köppen, Castilla y León presents a Mediterranean climate, characterized by long, cold winters (average January temperatures of 3 to 6 °C) and brief, warm summers (average temperatures of 19 to 22 °C). With an average annual rainfall of about 450 to 500 mm, which is especially visible in mountain ranges, there is very little rainfall throughout the summer. Moreover, distinct sub-climates can be identified because of the extensive area and orographic diversity of this region. A significant portion is in the subclimates of temperate with dry or temperate summer (Csb) or temperate with a dry season and temperate summer (Cfb), where the warmest month is typically below 22 °C but above 10 °C for at least five months. The sub-climate in several regions of the central plateau is categorized as either cold steppe (BSk), with average annual temperatures below 18 °C, or temperate with dry or hot summer (Csa), with summer temperatures above 22 °C. The climate at high elevations in mountainous regions is cold temperate, with dry summers (Dsb or Dsc) with average temperatures below 3 °C during the coldest months [21,22,23]. Furthermore, the geology of the region, dominated by sedimentary formations, plays a role in determining the pH and buffering capacity of surface waters.

A total of 126 samples from 42 sampling point were collected from 4 provinces (Salamanca (SW1-28), Valladolid (VW1-6), Zamora (ZW1-6) and Burgos (BW1-2)) in the autonomous community of Castilla y León (41°45′16″ N 4°46′55″ O) during the years 2022 and 2023, in order to detect the presence of potentially pathogenic FLA in water samples. Water collection in sterile bottles (1.5 L) was performed three different seasons: from September to November of 2022, where the temperature was approximately 12 °C (t1, autumn); from January to February of 2023, where the temperature was approximately 5 °C (t2, winter); and from May to June of 2023, where the temperature was approximately 16 °C (t3, late spring). These water samples that were taken approximately every 2 months during the study period correspond to the recreational fountain (36/126), tap from private home (33/126), river (18/126), swamp (12/126), irrigation water (15/126), and swimming pool (12/126) (Table 1).

### 2.2. Free-Living Amoeba Isolation

A total of 1.5 L of each water sample was filtered by a vacuum filtration system using a 0.45 μm pore size filter (Pall, Madrid, Spain). After that, the membrane filters were inverted onto 2% Non-Nutrient Agar (NNA) plates with a layer of heat-killed *E. coli*. The plates were cultured at room temperature and checked under an inverted microscope every day for amoebic growth for a maximum of 15 days. Once FLA was found on a plate, it was cloned by diluting it with fresh NNA until, if possible, a monoxenic culture was obtained. Before the use of molecular methods, isolated FLA were categorized according to Page’s criteria at the genus level based on their morphology [24].

### 2.3. DNA Extraction

For molecular characterization, DNA from positive samples was extracted from 1 to 2 milliliters of amoebic culture suspension. The monoxenic amoeba culture was washed with 4 mL of Page’s Amoeba Solution (PAS) and scraped with a glass staff to create the amoeba suspension. As previously described [25] the suspension was centrifuged and was then put straight into the Maxwell^®^ 16 tissue DNA purification kit sample cartridge (Promega, Madrid, Spain) in accordance with the manufacturer’s instructions. Extracted DNA yield and purity were quantified using the NanoDrop Lite Spectrophotometer (Fisher Scientific, Madrid, Spain).

### 2.4. PCR and Molecular Characterizations

Prior to being used as a template for PCR amplification of the 18S rRNA gene using various primer sets, extracted DNAs were kept at −20 °C. PCR was employed both as a confirmatory technique following preliminary morphological identification and as a molecular method for precise genus or species-level identification. The use of multiple primer sets was justified by the need to target different genera or families of free-living amoebae according to their morphological characteristics, allowing specific and efficient amplification: universal FLA-f 5′-CGCGGTAATTCCAGCTCCAATAGC-3′/FLA-r 5′-CAGGTTAAGGTCTCGTTCGTTAAC-3′ [26] and JDP-1f 5′-GGCCCAGATCGTTTACCGTGAA-3′ and JDP-2r 5′-TCTCACAAGCTGCTAGGGAGTCA-3′ for amoeba presenting morphology corresponding to *Acanthamoeba* spp. [27], Hv1227f 5′-TTACGAGGTCAGGACACTGT-3′ [28] and VermRV 5′-TGCCTCAAACTTCCATTCGC-3′ [29] for *V. vermiformis* and for the family Vahlkampfiidae we used these primers: VAHL1 5′-GTCTTCGTAGGTGAACCTGC-3′ and VAHL2 5′-CCGCTTACTGATATGCTTAA-3′ [30]. For all performed PCRs, amplification reactions were performed in a 50 μL mixture containing 80 ng DNA for FLA, 40 ng DNA for *Acanthamoeba* spp. and *V. vermiformis* and 60 ng DNA for VAHL, and the conditions for the PCRs shown in the table were established (Table 2). DNA is visualized with 2% agarose gel stained with Gel Red^®^. The positive PCR products obtained were sequenced by Macrogen Spain service. A homology search was performed with BLAST software within the GenBank database from the National Library of Medicine (NCBI).

### 2.5. Phylogenetic Analysis

To establish the genetic correlation among the isolates, a sequence alignment was performed using the MAFFT software version 7 with the accurate L-INS-i method [31] and trimAl for the removal of poorly aligned sites when necessary [32]. All evolutionary analyses were performed on RAxML v.8.2.10 using the Maximum Composite Likelihood method, which was utilized to compute the evolutionary distances, expressed in base substitutions per site [33]. RAxML used the GTRGAMMA substitution model for nucleotide alignments. To assess the robustness of the clusters, a bootstrap analysis was performed with 500 replicates, indicating the percentage of support next to each node. The tree was rooted with an external group.

## 3. Results

From the total of 126 samples for 42 sampling points, 69 water samples were positive for the presence of FLA on NNA plates (69/126; 54.76%). After analysis of the 18S rRNA gene (the DF3 region in the case of *Acanthamoeba*) 59 water samples (59/69; 85.51%) were positive in the PCR.

*Vermamoeba vermiformis* was the most abundantly isolated species in this study (35/69; 50.72%). *Acanthamoeba* spp. was the second most frequently found (16/69; 23.19%), with the T4 genotype being the most frequently detected (10/16; 62.5%) followed by the T2 (4/16; 25%), T3 (1/16; 6.25%) and T16 genotypes (1/16; 6.25%). Free-living amoebae of the Vahlkampfiidae family, including *Vahlkampfia avara* and *Naegleria pagei*, for example, were isolated to a lesser degree (Table S1).

The obtained nucleotide sequences are deposited in GenBank with the following accession numbers: PP853450-PP853471, PP872540-PP872541, PQ806969-PQ806983 and PV249448-PV249451. All of them presented ˃95% of homology with the available DNA sequences in this database.

### 3.1. Regional Variation

The results obtained show that, according to provinces: of the 126 total samples collected, 84 were from the province of Salamanca, 18 from the provinces of Zamora and Valladolid, and 6 from Burgos.

**Figure 2:**
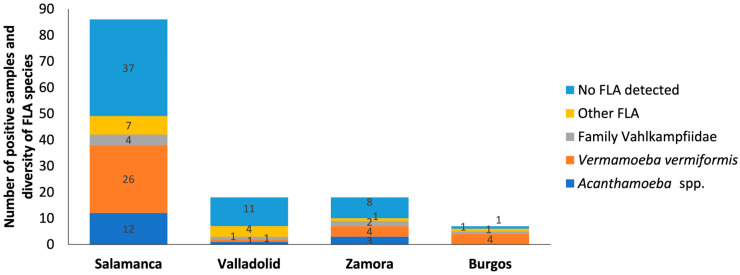
Distribution of free-living amoebae isolated in water samples for each province. The x-axis shows the provinces such as Salamanca, Valladolid, Zamora and Burgos. And the y-axis represents the number of positive samples and diversity of FLA species in the water samples. No FLA detected indicates the absence of free-living amoebae in the samples analyzed. Other FLA are amoebae not identified. Some of the samples contain more than one amoeba species.

**Figure 3:**
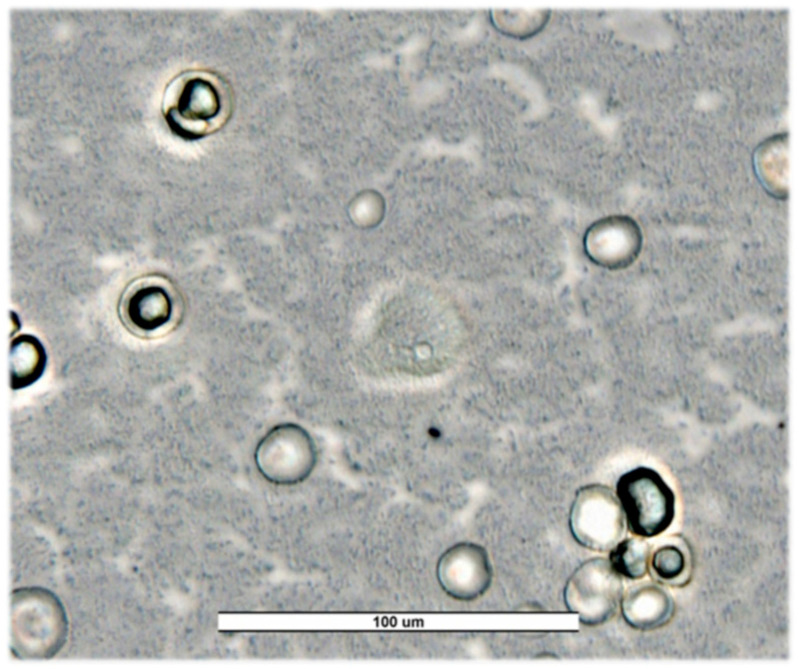
Trophozoites and cysts of *Naegleria americana* from Salamanca’s river (SW23) for first sampling. Image was obtained with an ECHO Revolution hybrid microscope (40×). Scale bar represents 100 μM.

**Salamanca.** Out of 84 samples tested, a prevalence of 55.95% (47/84) was obtained. *V. vermiformis* was the most predominant with 30.95% (26/84) (Figure 2). The genus *Acanthamoeba* was present in 14.29% of samples (12/84) and free-living amoebae of the family Vahlkampfiidae in 4.76% (4/84) (Figure 3). Other free-living amoebae were also identified in 8.33% of samples (7/84). Other FLA are those amoebae that are difficult to identify by the morphological approach. Salamanca showed higher diversity than the other provinces assessed. This large variety of FLA may be influenced by, among others, being the province with the largest number of samples.

**Figure 4:**
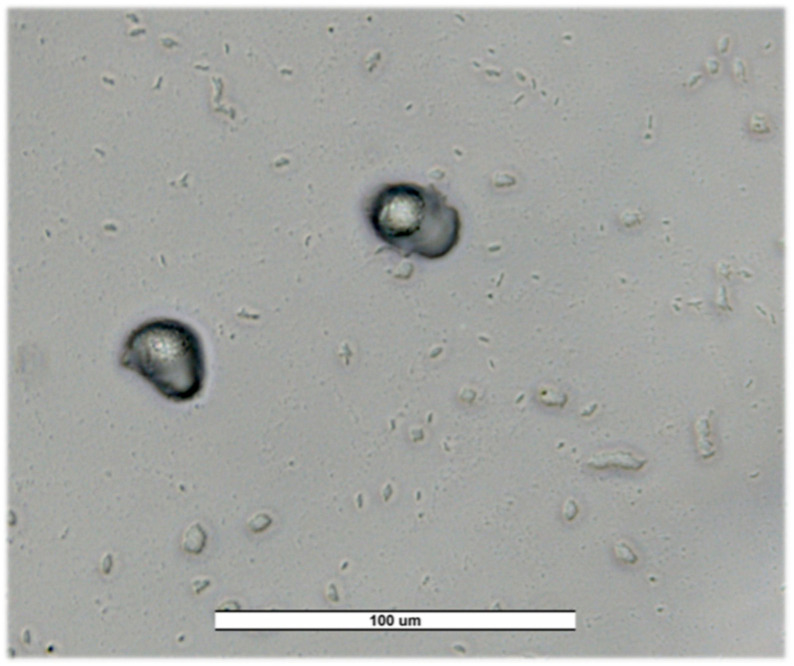
Trophozoites of *Vannella* spp. in a recreational fountain from Valladolid (VW5) for first sampling. Image was obtained with an ECHO Revolution hybrid microscope (40×). Scale bar represents 100 μM.

**Valladolid.** A prevalence of 38.89% (7/18) was obtained, with a predominance of other free-living amoebae (4/18; 22.22%), which could indicate the presence of less common species or the need for more advanced techniques for their identification (Figure 4). *V. vermiformis, Acanthamoeba* sp. T4 and *Naegleria* sp. were identified with a prevalence of 5.55% (1/18) for each of them.

**Figure 5:**
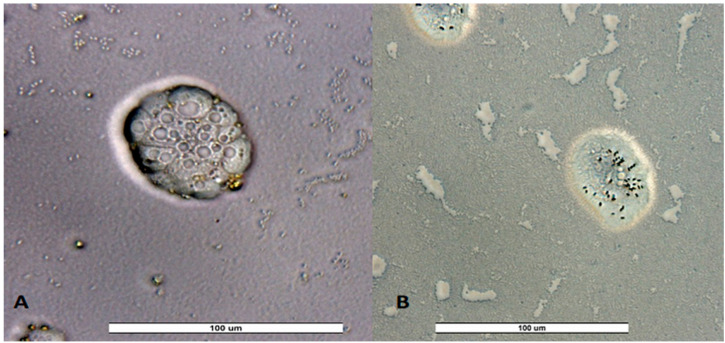
Trophozoites of unidentified amoeba from a recreational fountain sample from Valladolid VW5 (**A**) and Zamora ZW4 (**B**) for third sampling. Image was obtained with an ECHO Revolution hybrid microscope (40×). Scale bar represents 100 μM.

**Zamora**. A prevalence of 55.55% (10/18) was obtained. In this province, similar patterns to Salamanca were observed, *V. vermiformis* was the most commonly isolated species (4/18; 22.22%). Also, *Acanthamoeba* sp. T4 (3/18; 16.66%), *V. avara* (2/18; 11.11%) and other free-living amoebae (1/18; 5.55%) were found (Figure 5).

**Figure 6:**
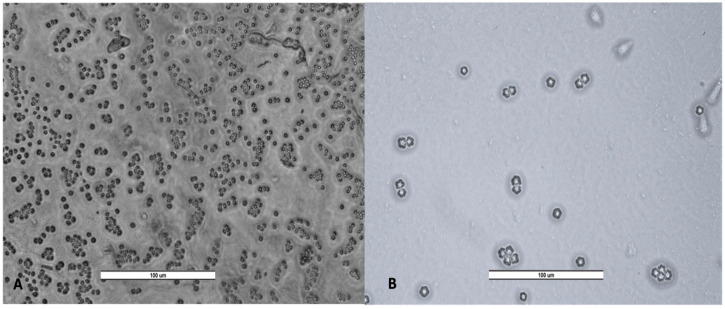
Cyst of *Vermamoeba vermiformis* (**A**) and trophozoites and cyst of *Acanthamoeba* spp. (**B**). Image was obtained with a LEICA DM500 microscope (20×). Scale bar represents 100 μM.

**Burgos.** A prevalence of 83.33% (5/6) was obtained, with *V. vermiformis* species standing out (4/6; 66.66%). In addition, *Vahlkampfia* sp. (1/6; 16.66%) and other free-living amoebae (1/6; 16.66%) were identified. While there was hardly any diversity in this study area, the prevalence of *V. vermiformis* was high, suggesting favorable local conditions for the growth of this amoeba (Figure 6). Therefore, its presence in this study emphasizes its ability to survive in different environmental conditions, underlining its adaptability.

### 3.2. Seasonal Variation

According to the season: Of the total of 126 samples distributed in 42 sampling points, 42 samples were taken for each of the stations (t1–t3), ensuring uniformity of the selected points and guaranteeing representativeness in each of the stations evaluated.

**Figure 7:**
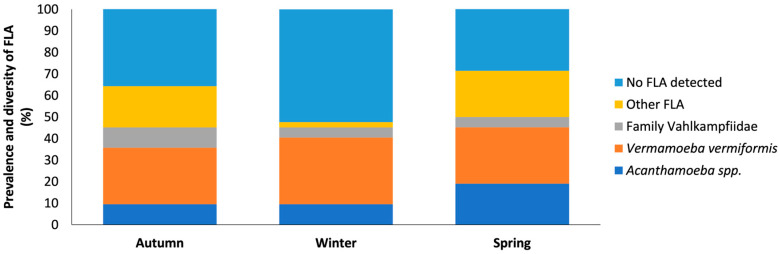
Distribution of free-living amoebae isolated in water samples for each season. The x-axis shows the season (t1–t3). And the y-axis represents the prevalence and diversity of FLA in the water samples. No FLA detected indicates the absence of free-living amoebae in the samples analyzed. Other FLA are amoebae not identified.

In the first sampling period (t1) a high prevalence and diversity of free-living amoebae was obtained in most provinces (24/42; 57.14%), with the species *V. vermiformis* (11/42) being the most abundant. However, the genus *Acanthamoeba*, species belonging to the family Vahlkampfiidae and other free-living amoebae were also found (Figure 7).

The second sampling period (t2) showed a decrease in the prevalence and diversity of free-living amoebae (20/42; 47.61%). However, a higher abundance of *V. vermiformis* (13/42) than of the genus *Acanthamoeba* was observed. On the other hand, from the family Vahlkampfiidae only one species of the genus *Vahlkampfia* was identified: *Vahlkampfia avara*.

In the third sampling period (t3), prevalence and diversity were higher than in the rest of the samplings (25/42; 59.52%). It should be noted that *V. vermiformis* was the most frequently identified species as seen in the previous samplings (11/42). However, a variety of free-living amoebae were isolated, including the genus *Acanthamoeba*, the genus *Vahlkampfia* and *Naegleria*, and other FLA.

Thus, in the first and third sampling period, a higher prevalence and diversity of free-living amoebae was observed in all provinces, except Zamora. In this province, unlike the general pattern, a higher prevalence was recorded in the second sampling (t2), suggesting a variation in environmental conditions or local factors that favored the increase in these amoebae in that period. In conclusion, our data shows a clear seasonal variation in FLA in the samples from the same sites taken in different seasons.

### 3.3. Influence of Type of Water Source

According to type of water: The different types of water analyzed (rivers, recreational fountain, irrigation water, swimming pools, swamps and drinking water) showed diverse patterns of prevalence and richness/diversity of FLA.

**Figure 8:**
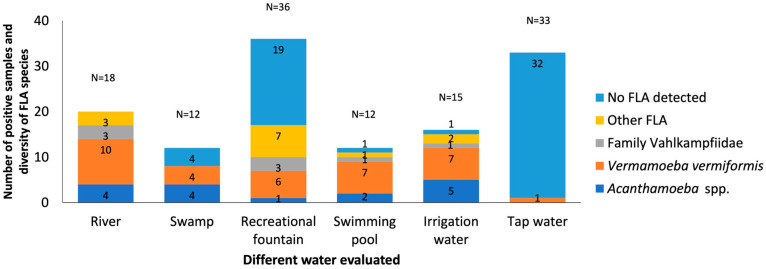
Distribution of free-living amoebae isolated in water samples for type of water. The x-axis shows the different types of water samples collected in different provinces such as Salamanca, Valladolid, Zamora, and Burgos. And the y-axis represents the number of positive samples and diversity of FLA species in the water samples. No FLA detected indicates the absence of free-living amoebae in the samples analyzed. Other FLA are amoebae not identified. Some of the samples contain more than one amoeba species.

Natural waters, as well as rivers and swamps, showed a remarkable prevalence of FLA; e.g., all river samples were positive for FLA culture (18/18; 100%), while from swamps only eight (8/12; 66.67%) exhibited a higher prevalence of *V. vermiformis, Acanthamoeba* spp., and less frequently, amoebae of the family Vahlkampfiidae (Figure 8).

Recreational fountains and swimming pools classified as recreational waters show results that differ from each other. Recreational fountains showed a 47.22% prevalence of FLA (17/36; 47.22%), with *V. vermiformis* being the most prevalent, possibly due to their greater exposure to the environment. In contrast, swimming pools showed a high prevalence of FLA (11/12; 91.66%) but lower diversity, but *V. vermiformis* was consistently present, suggesting its adaptability to chlorinated environments and potential failures in disinfection systems.

The irrigation water presents a prevalence of 93.33% (14/15). It was an important reservoir of *V. vermiformis* and *Acanthamoeba* spp., especially in agricultural areas. This finding highlights the importance of monitoring these sources, given their direct contact with crops and their role in public health.

The detection of FLA in only one drinking water sample (1/33; 3.03%) supports the high efficacy of the treatment systems in the region.

**Figure 9:**
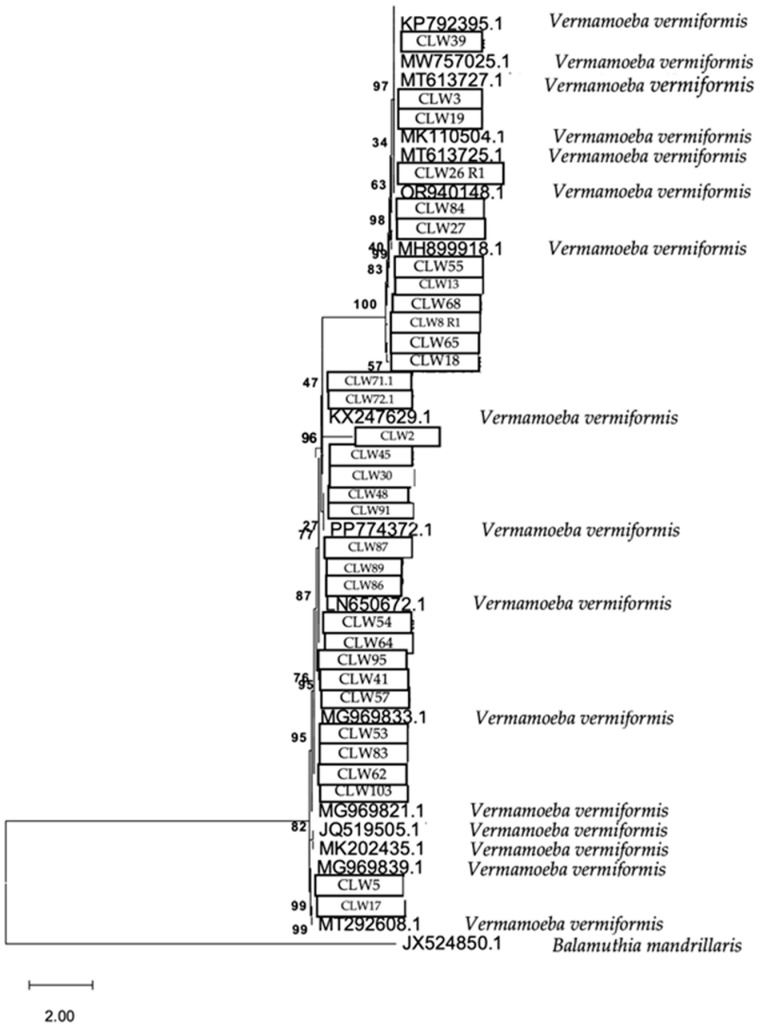
Phylogenetic connections among the species *Vermamoeba vermiformis* obtained from water samples in this study. The isolates obtained in this study are marked within boxes. Other species sequences were obtained from GenBank and their accession numbers are shown. The tree is computed using the Maximum Likelihood method and measured in the number of substitutions per site. The tree is rooted with *Balamuthia mandrillaris* as the outgroup. The percentage of replicate trees, in which the associated taxa are clustered together in the bootstrap test, is shown next to the branches (in bold). Scale bar = 2.00 substitutions/site.

**Figure 10:**
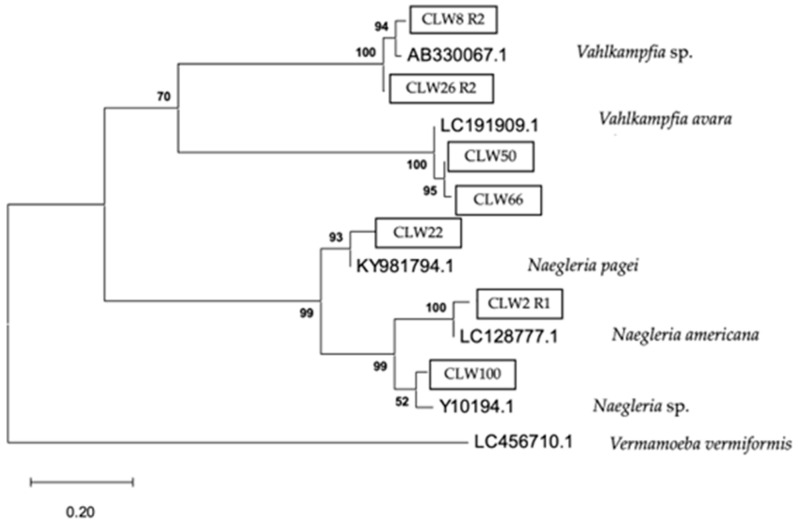
Phylogenetic connections among the family Vahlkampfiidae obtained from water samples in this study. The isolates obtained in this study are marked within boxes. Other species sequences were obtained from GenBank and their accession numbers are shown. The tree is computed using the Maximum Likelihood method and measured in the number of substitutions per site. The tree is rooted with *Vermamoeba vermiformis* as the outgroup. The percentage of replicate trees, in which the associated taxa are clustered together in the bootstrap test, is shown next to the branches (in bold). Scale bar = 0.20 substitutions/site.

**Figure 11:**
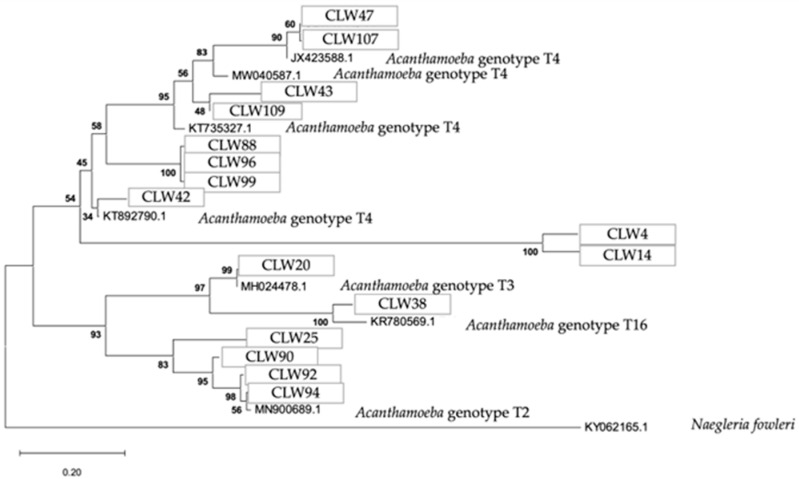
Phylogenetic connections among the genus *Acanthamoeba* obtained from water samples in this study. The isolates obtained in this study are marked within boxes. Other species sequences were obtained from GenBank and their accession numbers are shown. The tree is computed using the Maximum Likelihood method and measured in the number of substitutions per site. The tree is rooted with *Naegleria fowleri* as the outgroup. The percentage of replicate trees, in which the associated taxa are clustered together in the bootstrap test, is shown next to the branches (in bold). Scale bar = 0.20 substitutions/site.

The phylogenetic relationship of the FLA strains isolated in water samples is presented in Figure 9, Figure 10 and Figure 11, respectively.

The analytical procedure of the tree of the species *V. vermiformis* encompassed 1.972 aligned positions in the final dataset (Figure 9).

The analytical procedure of the tree of the family Vahlkampfiidae encompassed 818 aligned positions in the final dataset (Figure 10).

The analytical procedure of the tree of the genus *Acanthamoeba* encompassed 2.558 aligned positions in the final dataset (Figure 11).

## 4. Discussion

This manuscript shows the prevalence of the potentially pathogenic free-living amoebae in water from human related environments from Castilla y León, the largest region of the Iberian Peninsula and one of the largest territories of the European Union. FLA are distributed worldwide; i.e., they are cosmopolitan in nature [34]. Considering that these FLA represent a risk to both the environment and human health, different authors have been reporting their presence in a multitude of environments. However, there are currently few environmental studies on FLA epidemiology in Spain, and most of them have focused on the Canary Islands, especially on the island of Tenerife, which has been the most sampled island over time, reporting *V. vermiformis* species, the genera *Acanthamoeba* and *Naegleria* [35,36,37,38]. Previously, Lorenzo-Morales et al. (2005) focused on demonstrating the presence of *Acanthamoeba* in waters of Tenerife Island [36]. However, a study was conducted in later years that expanded the search for free-living amoebae on the same island, finding a greater diversity of FLA, including *Naegleria fultoni*, *Cercozoa* spp. and *Thecamoeba* spp., which are rare [16]. Compared to our current study, it is worth highlighting that the prevalence of the species *V. vermiformis* in the water samples was high, in line with what was observed on the island of Tenerife, Canary Islands. *V. vermiformis* is a ubiquitous and thermotolerant amoeba that is among the most common free-living amoebae. It has demonstrated pathogenic potential and has also been associated with harmful bacteria [39,40]. Previous studies have also reported the identification of this species in artificial recreational aquatic environments [41,42]. Contrasting these studies, a greater diversity of FLA was found in Castilla y León, as can be seen in Section 3; for example, *Vahlkampfia avara* was isolated, having been noted as a causative agent of keratitis [43]. Although *Acanthamoeba* spp. are the primary cause of amoebic keratitis, there have been various reported cases of coinfections of *Acanthamoeba* and *Vahlkampfia* genera and/or *Vahlkampfia*-related corneal injury [44,45,46]. This could be due to the completely different climate than that of the Canary Islands. In addition, the significant influence of the type of sample, province and season is evaluated.

Likewise, a brief investigation has recently been conducted over the course of a year in drinking water treatment plants (DWTPs), wastewater treatment plants (WWTPs), and other environmental waters, including reservoirs and rivers in the central region of Spain, which is located in the continental Mediterranean climate zone [47]. In comparison with this study, a fundamental difference lies in the type of sampled sources: while the study by Magnet and colleagues focused on treated waters within the urban water supply and sanitation systems, the present study focused on natural, recreational, and irrigation waters, that is, open ecosystems with greater direct contact with the environment. Therefore, while Magnet et al. concentrated on assessing health risks associated with treated water distribution systems, the present work provides valuable information on community-level risks related to the use of water for recreation and agriculture, highlighting the need to extend surveillance measures beyond urban and potable systems, into rural and semi-urban environments.

On the other hand, both studies agree in identifying the genus *Acanthamoeba*, particularly the T4 genotype, as one of the most abundant groups, reflecting its global prevalence and recognized pathogenic potential. *Acanthamoeba* is the genus best known to produce pathology in humans and it is also the one most commonly found in the environment [4]. This is relevant because of its association with serious human pathologies such as cerebral infections like granulomatous amoebic encephalitis (GAE), *Acanthamoeba* keratitis (AK), and less frequently, skin lesions [48,49].

Moreover, Magnet et al. observed seasonal variation, with a higher presence of FLA in spring and summer, which is consistent with the pattern observed in Castilla y León (maximum prevalence in spring and autumn). This seasonal behavior is related to optimal temperature conditions and nutrient availability that favor the trophic phase of amoebae.

Thus, the present work is the first to investigate these potential protozoa in the central north of the Iberian Peninsula (Autonomous Community of Castilla y León), providing new data that enrich the understanding of the biodiversity of these amoebae in aquatic environments in Spain. Moreover, our results contribute to the growing body of evidence supporting the need for continuous surveillance and standardized protocols for the detection of FLA, which are essential for the prevention of public health risks.

In this context, it is crucial to implement preventive measures aimed at reducing human exposure to these potentially pathogenic amoebae. Among the most important recommendations are the proper maintenance and disinfection of drinking water systems and swimming pools, strict control of water treatment conditions including the effective use of disinfectants such as chlorine and environmental monitoring to detect the presence of FLA at an early stage. Additionally, the public should be informed about the risks associated with contact with untreated or poorly maintained water, especially among vulnerable groups such as immunocompromised individuals.

Finally, considering that some FLA species can resist conventional treatments and proliferate in hospital and domestic environments, the implementation of specific management and control protocols in these settings is also a priority. The integration of molecular techniques alongside culture-based methods in environmental surveillance programs may enhance the detection and characterization of these amoebae, thus facilitating more informed decision-making in public health [41,50,51].

As mentioned, the results obtained in this study show great heterogeneity in the prevalence and diversity of FLA according on each province of Castilla y León, the sampling period, and the type of water. The lack of previous studies in the autonomous community of Castilla y León limits the comparison of historical data. However, the wide distribution of FLA evidences the need for more detailed investigations in other regions with similar climatic and geographical characteristics. Thus, the analysis by province revealed remarkable variations in the diversity and prevalence of FLA. On the other hand, the different types of water analyzed (rivers, recreational fountains, irrigation water, swimming pools, swamps and drinking water) showed diverse patterns of prevalence and richness of FLA, considering that natural waters, as well as rivers and swamps, showed a remarkable diversity of FLA, with a higher incidence of *V. vermiformis*, *Acanthamoeba* spp., and less frequently, amoebae of the family Vahlkampfiidae. High exposure to pollutants and environmental variations favors the proliferation of these species, especially during autumn and spring when climatic conditions are more moderate. In contrast, FLA were detected in just one drinking water sample, suggesting that the vast majority were negative and highlighting the efficiency of local treatment systems. However, past studies have indicated that certain FLA can survive the control of drinking water treatment plants, so continued monitoring is recommended [47,51].

Considering that the sampling was performed in three different periods (t1-t3), it was possible to identify seasonal patterns in the prevalence of FLA. On the one hand, the autumn and spring sampling period were the seasons with the highest prevalence of FLA. During these samplings, climatic conditions (moderate temperatures and higher humidity) are ideal for the growth of amoebae [52] as is the case with some groups of parasites [53]. In contrast, the second sampling showed a lower overall detection of FLA, which is attributed to lower temperatures that hinder trophozoite activity and favor their cyst state. These data corroborate/agree with studies described by Magnet et al. and Vingataramin et al. which show a similar seasonal pattern with peaks in autumn and spring [47,54]. There is even a report from a subtropical climate zone, Tulsa (Oklahoma, USA), that reported seasonality in the prevalence of *Acanthamoeba* in ponds in the United States [55].

According to the Köppen classification, the autonomous community of Castilla y León has a continental Mediterranean climate with certain subclimates in specific provinces due to geographical factors such as altitude and hydrography. This is a key factor in the distribution of free-living amoebae. Given that in temperate summer climates (Csb) established in the provinces of Salamanca, Zamora, and Burgos, the diversity of free-living amoebae, including *V. vermiformis*, *Acanthamoeba* spp. and genera of the family Vahlkampfiidae, is abundant. Also, the presence of large rivers, e.g., the Tormes River in Salamanca or the Duero River in Zamora, generates more humid microclimates that favor the persistence of FLA. In contrast, in more extreme climates such as BSk, which mainly occurs in Valladolid, there is a low diversity of FLA with a predominance of species resistant to environmental stress conditions such as *Acanthamoeba* spp. since low winter temperatures and lower humidity reduce trophozoite activity and favor the formation of resistant cysts.

Overall, the results of this study show the influence of the climate of each province, according to the Köppen classification, and the type of water on the distribution and abundance of FLA in Castilla y León. The combination of temperate and more extreme climates within the region determines the diversity and adaptability of these amoebae, evidencing the need to implement continuous surveillance and control measures, especially in recreational and agricultural waters, to protect public health against emerging pathogens such as *Acanthamoeba* spp. and *V. vermiformis*.

It is essential to emphasize that the results obtained in this study do not depend exclusively on environmental or seasonal factors, but are deeply influenced by several methodological aspects, such as sampling design, isolation protocol, incubation temperature used during primary culture, and strain selection procedure. These factors can significantly condition the recovery of FLA, favoring especially those species that show a higher growth capacity under laboratory conditions, or that are present in the sample at higher concentrations. In particular, the incubation temperature is decisive for the activation of the trophic state of the amoebae, which directly influences the success of the culture and, therefore, the detection of the species present.

## 5. Conclusions

This study provides evidence of the environmental of FLA in Castilla y León, highlighting the importance of implementing epidemiological surveillance strategies in susceptible water sources. Given the geographical and seasonal variability of FLA, it is essential to develop specific control policies for each type of water source, with special attention to recreational and agricultural waters, to reduce the risks associated with these potentially pathogenic protozoa.

## Figures and Tables

**Table 1:** Location of environmental water samples analyzed for FLA detection in the autonomous community of Castilla y León.

*Sample*	*Locality*	*Coordinates*	*Province*	*Water Type*
SW1	Salamanca	40.966204, −5.668706	Salamanca	Recreational fountain
SW2	Salamanca	40.967639, −5.651176	Salamanca	Recreational fountain
SW3	Salamanca	40.955600, −5.668074	Salamanca	River
SW4	Salamanca	40.958195, −5.669783	Salamanca	Tap water
SW5	Salamanca	40.968051, −5.658020	Salamanca	Recreational fountain
SW6	Salamanca	40.967422, −5.667352	Salamanca	Recreational fountain
SW7	Salamanca	40.964468, −5.681959	Salamanca	Tap water
SW8	Salamanca	40.976289, −5.661977	Salamanca	Recreational fountain
SW9	Salamanca	40.965070, −5.678534	Salamanca	Tap water
SW10	Aldeatejada	40.946595, −5.681442	Salamanca	Irrigation water
SW11	Aldeatejada	40.945903, −5.681217	Salamanca	Swimming pool
SW12	Aldeatejada	40.945903, −5.681217	Salamanca	Tap water
SW13	Galindo y Perahuy	40.924286 −5.804917	Salamanca	Swimming pool
SW14	Galindo y Perahuy	40.926787, −5.807606	Salamanca	Irrigation water
SW15	Galindo y Perahuy	40.924027, −5.808318	Salamanca	Swimming pool
SW16	Galindo y Perahuy	40.925187, −5.805347	Salamanca	Tap water
SW17	Trabanca	41.231362, −6.386124	Salamanca	Swimming pool
SW18	Trabanca	41.231895, −6.383861	Salamanca	Tap water
SW19	Trabanca	41.232983, −6.384409	Salamanca	Recreational fountain
SW20	Almendra	41.273762, −6.321856	Salamanca	Swamp
SW21	Espadaña	41.131510, −6.361370	Salamanca	Swamp
SW22	Almendra	41.270194, −6.334739	Salamanca	Swamp
SW23	Miranda de Azán	40.886026, −5.684305	Salamanca	River
SW24	Miranda de Azán	40.875737, −5.683251	Salamanca	Irrigation water
SW25	Miranda de Azán	40.886327, −5.682139	Salamanca	Tap water
SW26	Miranda de Azán	40.871145, −5.682950	Salamanca	Irrigitation water
SW27	Miranda de Azán	40.874163, −5.683229	Salamanca	Irrigation water
SW28	Miranda de Azán	40.888639, −5.683626	Salamanca	River
VW1	Valladolid	41.646338, −4.744723	Valladolid	Tap water
VW2	Valladolid	41.643510, −4.715008	Valladolid	Tap water
VW3	Valladolid	41.657431, −4.733970	Valladolid	River
VW4	Valladolid	41.644816, −4.730373	Valladolid	Recreational fountain
VW5	Valladolid	41.645413, −4.729856	Valladolid	Recreational fountain
VW6	Valladolid	41.579598, −4.661287	Valladolid	Swamp
ZW1	Zamora	42.003143, −5.677092	Zamora	Tap water
ZW2	Zamora	41.995946, −5.682736	Zamora	Recreational fountain
ZW3	Zamora	41.507055, −5.740209	Zamora	Recreational fountain
ZW4	Zamora	41.506300, −5.740005	Zamora	Recreational fountain
ZW5	Zamora	41.496178, −5.754736	Zamora	River
ZW6	Zamora	41.495268, −5.741160	Zamora	Tap water
BW1	Burgos	42.337932, −3.705546	Burgos	River
BW2	Burgos	42.339900, −3.698603	Burgos	Recreational fountain

**Table 2:** PCR conditions for the different primers.

Primer Sets	PCR’s Conditions				
	*Initiation*	*Denaturation*	*Annealing*	*Primer Extension*	*Extension*
Hv1227F/VermR	95 °C—5 min	95 °C—30 min	50 °C—30 min	72 °C—30 min	72 °C—7 min
		35 Cycles			
JDP1/JDP2	95 °C—2 min	95 °C—30 min	50 °C—30 min	72 °C—30 min	72 °C—7 min
		35 Cycles			
FLAf/FLAr	95 °C—2 min	95 °C—30 min	55 °C—30 min	72 °C—30 min	72 °C—7 min
		40 Cycles			
VAHL1/VAHL2	95 °C—5 min	95 °C—30 min	55 °C—30 min	72 °C—30 min	72 °C—7 min
		35 Cycles			

## Data Availability

Data are contained within the article and Supplementary Materials.

## Supplementary Materials

The following supporting information can be downloaded at: https://www.mdpi.com/article/10.3390/pathogens14070637/s1, Table S1: Additional details, results, including tables showing the presence of free-living amoebae in each of the water samples from Castilla y León for the three sampling periods.
