# Supplementary material for: High Diversity and Prevalence of Potentially Pathogenic Free-Living Amoebae in Water Sources from Castilla y León, Spain
Source: Pathogens. 2025 Jun 25;14(7):637. doi: 10.3390/pathogens14070637 (PMC12300931; doi:10.3390/pathogens14070637)
Supplement: Supplementary file 1 [file pathogens-14-00637-s001.zip › pathogens-3656556-supplementary.pdf]

Table S1. FLA species isolated from the evaluated water sources from the autonomous community of Castilla y León in different sampling (NNA: FLA growth in non-nutrient agar culture; PCR: FLA detection by PCR; homology (%) related to NCBI Database sequence).

Water samples								
**Sampling**	**Sample code**	**Province**	**Water type**	**NNA**	**PCR**	**Genus/species**	**Genbank code**	**Homology (%)**
T1	SW1	Salamanca	Recreational fountain	-				
	SW2	Salamanca	Recreational fountain	+	-	Unidentified amoeba		
	SW3	Salamanca	River	+	-	Unidentified amoeba		
	SW4	Salamanca	Tap water	-				
	SW5	Salamanca	Recreational fountain	+	-	Unidentified amoeba		
	SW6	Salamanca	Recreational fountain	-				
	SW7	Salamanca	Tap water	-				
	SW8	Salamanca	Recreational fountain	-				
	SW9	Salamanca	Tap water	-				
	SW10	Salamanca	Irrigation water	+	+	*Vermamoeba vermiformis*	CLW8 R1	≥ 95%
						Vahlkampfia sp.	CLW8 R2	
	SW11	Salamanca	Swimming pool	+	+	*Acanthamoeba palestinensis* T2	CLW25	≥ 95%
	SW12	Salamanca	Tap water	-				
	SW13	Salamanca	Swimming pool	+	+	*Naegleria pagei*	CLW22	≥ 95%
	SW14	Salamanca	Irrigation water	-				
	SW15	Salamanca	Swimming pool	+	-	Unidentified amoeba		
	SW16	Salamanca	Tap water	-				
	SW17	Salamanca	Tap water	-				
	SW18	Salamanca	Swimming pool	+	+	*Vermamoeba vermiformis*	CLW19	≥ 95%
	SW19	Salamanca	Recreational fountain	+	+	*Vermamoeba vermiformis*	CLW21	≥ 95%
	SW20	Salamanca	Swamp	+	+	*Acanthamoeba* sp. T3	CLW20	≥ 95%
	SW21	Salamanca	Swamp	+	+	*Vermamoeba vermiformis*	CLW17	≥ 95%
	SW22	Salamanca	Swamp	+	+	*Vermamoeba vermiformis*	CLW18	≥ 95%

	SW23	Salamanca	River	+	+	*Vermamoeba vermiformis*	CLW2 R2	≥ 95%
						Naegleria americana	CLW2 R1	
	SW24	Salamanca	Irrigation water	+	+	*Vermamoeba vermiformis*	CLW3	≥ 95%
	SW25	Salamanca	Tap water	-				
	SW26	Salamanca	Irrigation water	+	+	*Acanthamoeba* sp. T4	CLW4	≥ 95%
	SW27	Salamanca	Irrigation water	+	+	*Vermamoeba vermiformis*	CLW5	≥ 95%
	SW28	Salamanca	River	+	-	Unidentified amoeba		
	VW1	Valladolid	Tap water	-				
	VW2	Valladolid	Tap water	-				
	VW3	Valladolid	River	+	+	*Amebozoa* sp.	CLW11	≥ 95%
	VW4	Valladolid	Recreational fountain	+	+	*Amebozoa* sp.	CLW10	≥ 95%
	VW5	Valladolid	Recreational fountain	+	+	*Vannella* sp.	CLW9	≥ 95%
	VW6	Valladolid	Swamp	-				
	ZW1	Zamora	Tap water	-				
	ZW2	Zamora	Recreational fountain	-				
	ZW3	Zamora	Recreational fountain	-				
	ZW4	Zamora	Recreational fountain	+	+	*Vermamoeba vermiformis*	CLW13	≥ 95%
	ZW5	Zamora	River	+	+	*Acanthamoeba* sp. T4	CLW14	≥ 95%
	ZW6	Zamora	Tap water	-				≥ 95%
	BW1	Burgos	River	+	+	*Vermamoeba vermiformis*	CLW26 R1	≥ 95%
						Vahlkamfpia sp.	CLW26 R2	
	BW2	Burgos	Recreational fountain	+	+	*Vermamoeba vermiformis*	CLW27	≥ 95%
T2	SW1	Salamanca	Recreational fountain	-				
	SW2	Salamanca	Recreational fountain	-				
	SW3	Salamanca	River	+	+	*Vermamoeba vermiformis*	CLW30	≥ 95%
	SW4	Salamanca	Tap water	-				
	SW5	Salamanca	Recreational fountain	-				
	SW6	Salamanca	Recreational fountain	-				
	SW7	Salamanca	Tap water	-				

	SW8	Salamanca	Recreational fountain	-				
	SW9	Salamanca	Tap water	-				
	SW10	Salamanca	Irrigation water	+	+	*Acanthamoeba* sp. T16	CLW38	≥ 95%
	SW11	Salamanca	Swimming pool	+	+	*Vermamoeba vermiformis*	CLW39	≥ 95%
	SW12	Salamanca	Tap water	-				
	SW13	Salamanca	Swimming pool	+	+	*Vermamoeba vermiformis*	CLW41	≥ 95%
	SW14	Salamanca	Irrigation water	+	+	*Acanthamoeba* sp. T4	CLW42	≥ 95%
	SW15	Salamanca	Swimming pool	+	+	*Acanthamoeba castellani* T4	CLW43	≥ 95%
	SW16	Salamanca	Tap water	-				
	SW17	Salamanca	Tap water	-				
	SW18	Salamanca	Swimming pool	+	+	*Vermamoeba vermiformis*	CLW45	≥ 95%
	SW19	Salamanca	Recreational fountain	-				
	SW20	Salamanca	Swamp	+	+	*Acanthamoeba* sp. T4	CLW47	≥ 95%
	SW21	Salamanca	Swamp	+	+	*Vermamoeba vermiformis*	CLW48	≥ 95%
	SW22	Salamanca	Swamp	-				
	SW23	Salamanca	River	+	+	*Vahlkampfia avara*	CLW50	≥ 95%
	SW24	Salamanca	Irrigation water	+	-	Unidentified amoeba		
	SW25	Salamanca	Tap water	-				
	SW26	Salamanca	Irrigation water	+	+	*Vermamoeba vermiformis*	CLW53	≥ 95%
	SW27	Salamanca	Irrigation water	+	+	*Vermamoeba vermiformis*	CLW54	≥ 95%
	SW28	Salamanca	River	+	+	*Vermamoeba vermiformis*	CLW55	≥ 95%
	VW1	Valladolid	Tap water	-				
	VW2	Valladolid	Tap water	-				
	VW3	Valladolid	River	+	+	*Vermamoeba vermiformis*	CLW57	≥ 95%
	VW4	Valladolid	Recreational fountain	-				
	VW5	Valladolid	Recreational fountain	-				
	VW6	Valladolid	Swamp	-				
	ZW1	Zamora	Tap water	+	+	*Vermamoeba vermiformis*	CLW64	≥ 95%

	ZW2	Zamora	Recreational fountain	+	+	*Vermamoeba vermiformis*	CLW65	≥ 95%
	ZW3	Zamora	Recreational fountain	+	+	*Vahlkampfia avara*	CLW66	≥ 95%
	ZW4	Zamora	Recreational fountain	-				
	ZW5	Zamora	River	+	+	*Vermamoeba vermiformis*	CLW68	≥ 95%
	ZW6	Zamora	Tap water	-				
	BW1	Burgos	River	+	+	*Vermamoeba vermiformis*	CLW62	≥ 95%
	BW2	Burgos	Recreational fountain	-				
T3	SW1	Salamanca	Recreational fountain	-				
	SW2	Salamanca	Recreational fountain	+	**+**	*Vermamoeba vermiformis*	CLW71.1	≥ 95%
	SW3	Salamanca	River	+	**+**	*Vermamoeba vermiformis*	CLW72.1	≥ 95%
	SW4	Salamanca	Tap water	-				
	SW5	Salamanca	Recreational fountain	-				
	SW6	Salamanca	Recreational fountain	-				
	SW7	Salamanca	Tap water	-				
	SW8	Salamanca	Recreational fountain	-				
	SW9	Salamanca	Tap water	-				
	SW10	Salamanca	Irrigation water	+	-	Unidentified amoeba		
	SW11	Salamanca	Swimming pool	-				
	SW12	Salamanca	Tap water	-				
	SW13	Salamanca	Swimming pool	+	+	*Vermamoeba vermiformis*	CLW82	≥ 95%
	SW14	Salamanca	Irrigation water	+	+	*Vermamoeba vermiformis*	CLW83	≥ 95%
	SW15	Salamanca	Swimming pool	+	+	*Vermamoeba vermiformis*	CLW84	≥ 95%
	SW16	Salamanca	Tap water	-				
	SW17	Salamanca	Tap water	-				
	SW18	Salamanca	Swimming pool	+	+	*Vermamoeba vermiformis*	CLW86	≥ 95%
	SW19	Salamanca	Recreational fountain	+	+	*Vermamoeba vermiformis*	CLW87	≥ 95%
	SW20	Salamanca	Swamp	+	+	*Acanthamoeba* sp. T4	CLW88	≥ 95%

	SW21	Salamanca	Swamp	+	+	*Vermamoeba vermiformis*	CLW89	≥ 95%
	SW22	Salamanca	Swamp	+	+	*Acanthamoeba palestinensis* T2	CLW90	≥ 95%
	SW23	Salamanca	River	+	+	*Vermamoeba vermiformis*	CLW91	≥ 95%
	SW24	Salamanca	Irrigation water	+	+	*Acanthamoeba palestinensis* T2	CLW92	≥ 95%
	SW25	Salamanca	Tap water	-				
	SW26	Salamanca	Irrigation water	+	+	*Acanthamoeba* sp. T2	CLW94	≥ 95%
	SW27	Salamanca	Irrigation water	+	+	*Vermamoeba vermiformis*	CLW95	≥ 95%
	SW28	Salamanca	River	+	+	*Acanthamoeba* sp. T4	CLW96	≥ 95%
	VW1	Valladolid	Tap water	-				
	VW2	Valladolid	Tap water	-				
	VW3	Valladolid	River	+	+	*Acanthamoeba* sp. T4	CLW99	≥ 95%
	VW4	Valladolid	Recreational fountain	+	+	*Naegleria* sp.	CLW100	≥ 95%
	VW5	Valladolid	Recreational fountain	+	-	Unidentified amoeba		
	VW6	Valladolid	Swamp	-				
	ZW1	Zamora	Tap water	-				
	ZW2	Zamora	Recreational fountain	+	+	*Vahlkampfia avara*	CLW106	≥ 95%
	ZW3	Zamora	Recreational fountain	+	+	*Acanthamoeba* sp. T4	CLW107	≥ 95%
	ZW4	Zamora	Recreational fountain	+	-	Unidentified amoeba		
	ZW5	Zamora	River	+	+	*Acanthamoeba* sp. T4	CLW109	≥ 95%
	ZW6	Zamora	Tap water	-				
	BW1	Burgos	River	+	+	*Vermamoeba vermiformis*	CLW103	≥ 95%
	BW2	Burgos	Recreational fountain	+	-	Unidentified amoeba		
